# Vitamin D deficiency, supplementation, and colorectal cancer outcomes: interactions with obesity and risk profiles

**DOI:** 10.3389/fmed.2025.1657534

**Published:** 2025-09-12

**Authors:** Basmalah Naji, Menatalla Eltawil, Najla Nemer, Omer Abdelazim, Jayaditya D. Patil, Salim Fredericks

**Affiliations:** ^1^School of Medicine, RCSI – Medical University of Bahrain, Al Sayh, Bahrain; ^2^Department of Surgery, Yale, CT, United States; ^3^Department of Biochemistry, School of Medicine, RCSI – Medical University of Bahrain, Al Sayh, Bahrain

**Keywords:** vitamin D, colorectal cancer, VDR polymorphisms, vitamin D supplementation, obesity, cancer prognosis

## Abstract

**Background:**

Vitamin D deficiency, colorectal cancer, and tumor progression are increasingly linked in recent research. Beyond its well-established roles in bone metabolism and immune regulation, vitamin D has emerged as a potential modulator of cancer prevention and prognosis, particularly in colorectal cancer, where deficiency may worsen outcomes.

**Purpose:**

Vitamin D is critical in the prevention and prognosis of colorectal cancer, such as colorectal adenocarcinoma. This review aims to explore the impact of Vitamin D deficiency on colorectal cancer progression and assess the role of vitamin D supplementation in improving outcomes.

**Methods:**

A narrative review was conducted, utilizing five databases: PubMed, Medline Plus, ScienceDirect, Scopus, and Google Scholar, focusing on human studies published in the last 15 years (from 2012 to 2025). Priority was given to primary studies like randomized controlled trials and cohort studies, while systematic reviews were included for broader context. Exclusion criteria included animal studies, non-English papers, and non-peer-reviewed content.

**Results:**

The review synthesizes evidence from 33 primary studies and 16 high-quality reviews. Findings indicate that vitamin D supplementation may enhance prognosis by influencing serum levels, immune modulation, and gut microbiota. However, clinical trials results are mixed, particularly concerning optimal dosing, genetic variability, and factors like obesity.

**Discussion:**

Vitamin D supplementation shows promise in improving colorectal cancer prognosis, but further research is necessary to refine dosing strategies and develop personalized therapies tailored to individual patient needs.

## 1 Introduction

Vitamin D is a fat-soluble vitamin known for its roles in calcium homeostasis, immune function, and cellular regulation. Deficiency in vitamin D is a widespread global issue affecting approximately 1 billion people globally and has been linked to various chronic diseases (1). Up to 90% of active vitamin D (1,25-(OH)₂D₃) is synthesized endogenously through cutaneous exposure to ultraviolet B (UVB) radiation, while dietary sources and supplements contribute a much smaller share (2). In recent years, the role of Vitamin D in the pathophysiology and prognosis of colorectal cancer (CRC) has garnered increasing attention, particularly due to its involvement in processes such as inflammation, cell proliferation, and apoptosis.

While early detection and therapeutic interventions have improved outcomes, prognosis in advanced CRC disease remains poor. To tackle that, there has been a growing interest in understanding how micronutrients like vitamin D might potentially influence cancer progression and therapeutic response.

This review aims to explore the current body of primary evidence and reviews regarding vitamin D supplementation and its effects on CRC outcomes. We examine proposed biological mechanisms, clinical trial results, and potential confounders such as obesity and genetic factors. The objective is to analyze data presented in original research studies and some reviews to clarify the prognostic significance of vitamin D in CRC.

## 2 Methods

This review is a narrative synthesis of existing literature examining the relationships between vitamin D supplementation, obesity, and colorectal cancer (CRC) outcomes. We searched five databases—PubMed, MedlinePlus, ScienceDirect, Scopus, and Google Scholar—for human studies published in English over the past 15 years (from 2012 to 2025). Keywords included combinations of *“vitamin D,” “colorectal cancer,” “supplementation,” “obesity,”* and *“VDR polymorphisms.”* Priority was given to clinical studies (e.g., randomized controlled trials, cohort studies, case–control) and high-quality systematic reviews or meta-analyses.

We predominantly excluded non-peer-reviewed articles, non-English publications, animal studies, and studies lacking relevance to the clinical aspects of vitamin D in CRC. Based on thematic relevance rather than formal screening, a total of 41 studies were included: 18 primary studies and 23 systematic reviews/meta-analyses. See Table 1 for a systematic summary of the primary studies in our review.

**Table 1:** Extended methodological overview of studies on Vitamin D supplementation and CRC outcomes.

No.	Authors and year	Study type	Population and sample size	Vitamin D dose or serum level	Main findings	Relevance to CRC outcome
1	Zhu et al. (2018) (6)	Cohort study	3,818 participants (2,166 females)	Serum 25(OH)D concentration was 60.6 ± 18.0 nmol/L	For colorectal cancer, lower circulating 25(OH)D was associated with significantly higher risk compared with the middle group (covariate-adjusted HR 1.62, 95% CI 1.04, 2.53).	Lower 25(OH)D levels were associated with increased risk of colorectal and breast cancer, but not overall cancer risk.
2	Chiang et al. (2023) (7)	Cross-sectional study	1,306 participants	No specific dose provided, the paper investigated the impact of different vitamin D levels on the risk of colorectal polyps.	Results showed that the prevalence of 25(OH)-vitamin D deficiency (≦ 20 ng/mL) and colorectal polyps was 21.21 and 40.89%, respectively.	Study revealed that Vitamin D deficiency was significantly associated with the risk of colorectal polyps, especially in adults over 50 years old and women.
3	Latacz et al. (2021) (8)	Case - Control study	103 patients diagnosed with CRC (61 men and 42 women) and 109 healthy people	The study shows possible association of the vitamin D receptor (VDR) polymorphism with CRC susceptibility.	None of the single nucleotide polymorphisms (SNPs) individually increased or decreased the risk of CRC.	A creation of a relevant SNP’s panel might contribute to the identification of the groups that are at the greatest risk of CRC.
4	Onali et al. (2025) (9)	RCT	43 healthy adults were randomized	There was no mention of Vitamin D serum levels of specific dosage	In comparison to the Meat group, berry consumption resulted in higher fecal concentrations of p-coumaric and protocatechuic acids and lower viability of fecal water (FW) -treated CV1-P fibroblastoma and human colon adenocarcinoma	Berry consumption provided protective nutrients and mitigated potentially unfavourable gut microbiota changes seen in the Meat group, increased fecal polyphenol metabolites, and reduced viability of FW-treated colon adenocarcinoma cells, collectively suggesting that berries may protect against colorectal cancer development.
5	McCullough et al. (2019) (10)	Prospective cohort	5,706 colorectal cancer case participants and 7,107 control participants	The study measured Study-specific relative risks (RRs) for prediagnostic season-standardized 25(OH)D concentrations.	For each 25 nmol/L increment in circulating 25(OH)D, colorectal cancer risk was 19% lower in women (RR = 0.81, 95% CI = 0.75 to 0.87) and 7% lower in men (RR = 0.93, 95% CI = 0.86 to 1.00) (two-sided Pheterogeneity by sex = 0.008)	Compared with the lower range of sufficiency for bone health (50- < 62.5 nmol/L), deficient 25(OH)D (<30 nmol/L) was associated with 31% higher colorectal cancer risk.
6	Väyrynen et al. (2016) (13)	Cohort	117 CRC patients and 86 controls	The study measured different vitamin D serum levels in the 117 patients and 86 outcomes and were analyzed with disease outcome.	The patients had lower serum 25(OH)D levels compared to the controls. In addition, patients operated in summer or autumn had higher serum 25(OH)D levels	Serum 25(OH)D levels inversely correlated with several systemic inflammatory markers, e.g., serum C reactive protein, but did not associate with prognosis
7	Wesselink et al. (2020) (17)	Cross-sectional study	1,201 newly-diagnosed stage I–III CRC patients	Measuring different 25(OH)D3 serum concentrations in CRC patients at diagnosis and six months later.	Vitamin D intake from diet or supplements, Use of calcium supplements, BMI and disease stage were associated with 25(OH)D3 levels at both time points. Six months after diagnosis, gender and having received chemo- and/or radiotherapy were also associated with 25(OH)D3 levels.	In conclusion, vitamin D supplement Use and treatment appear to be important determinants of 25(OH)D3 levels during the first six months after CRC diagnosis.
8	Gibbs et al. (2021) (24)	RCT	2,259 participants	1,000 IU	Among those with the DBP2 isoform (rs4588*AC or AA), the RRs (95% CI) for adenoma recurrence were 0.84 (0.72–1.00) with vitamin D3 relative to no vitamin D3	Individuals with the DBP2 isoform-encoding rs4588*A allele may particularly benefit from vitamin D3 and/or calcium supplementation for colorectal adenoma prevention.
9	Sutherland et al. (2020) (25)	Retrospective screening-based, Cross-sectional study	1,409 patients	(600 IU)	Meeting the recommended daily intake (RDI) of vitamin D is protective against HRAPs	This study suggests that adequate vitamin D supplementation reduces the occurrence of colorectal polyps in high-latitude locations.
10	Bellerba et al. (2022) (26)	RCT	74 CRC patients	2000 IU/day vitD	Those achieving Vitamin D sufficiency (25(OH)D ≥ 30 ng/mL) had lower post-treatment abundances (*p* = 0.05).	Vitamin D supplementation may contribute shaping the gut microbiota and the microbiota may partially mediate the effect of supplementation on 25(OH)D
11	Fuchs et al. (2017) (27)	RCT	1,016 patients with stage III colon cancer	27.6 ng ml − 1	Patients in the highest quintile of predicted 25(OH)D score had an adjusted hazard ratio (HR) for colon cancer recurrence or mortality (DFS) of 0.62 (95% confidence interval [CI], 0.44–0.86), compared with those in the lowest quintile (Ptrend = 0.005)	Higher predicted 25(OH)D levels after a diagnosis of stage III colon cancer may be associated with decreased recurrence and improved survival
12	Wesselink et al. (2020) (28)	Prospective cohort	1,169 newly diagnosed stage I–III CRC patients	≥50 nmol/L	We observed the lowest risk of all-cause mortality in patients with sufficient vitamin D concentrations (≥50 nmol/L) and a high magnesium intake (median split) (HR: 0.53; 95% CI: 0.31, 0.89) compared with patients who were vitamin D deficient (<50 nmol/L) and had a low magnesium intake	Our findings suggest that the presence of an adequate status of 25(OH)D3 in combination with an adequate magnesium intake is essential in lowering the risk of mortality in CRC patients,
13	Messaritakis et al. (2020) (30)	Prospective study	397 patients	The study measured the different vitamin D levels among the patients.	The results of the present study highlight the significant role of VDR polymorphisms in carcinogenesis, disease progression and patients’ survival.	Higher frequencies of the tt, aa, ff and bb genotypes were detected in metastatic CRC patients compared to stage II/III patients, emphasizing the role of these polymorphisms in CRC progression and in patients’ overall survival.
14	Zhu et al. (2005) (3)	RCT, Animal study	4-week-old IL-10 KO mice used; however, the study did not specify the exact number of mice used in the study	No exact mention of the amount of vitamin D serum levels used	The study showed for the first time that both 1,25D3 and dietary calcium independently and additively suppressed IBD in IL-10 KO mice. High dietary calcium and 1,25D3 treatment together reduced SI/BW and LI/BW ratios by 40 and 48%, respectively	These findings support our investigation of the TaqI polymorphism in the VDR gene and CRC risk by showing that VDR signaling modulates intestinal inflammation via the TNF-*α* pathway. Since chronic inflammation like IBD is a known CRC risk factor, variations in VDR, such as TaqI, may influence individual CRC susceptibility. The animal model highlights VDR’s protective role and the plausibility of its genetic impact on CRC.
15	Rong et al. (2023) (33)	In silico plus *in vivo* experimental preclinical study	51 targets of vitamin D3	The relevant targets for vitamin D3 and CRC were obtained from the database of drug and disease targets, respectively.	This study will provide more theoretical support for vitamin D3 to reduce the incidence of CRC and is important to explore more pharmacological effects of vitamin D3.	The results suggest that vitamin D3 plays a key role in the prevention of CRC through core targets, PI3K-Akt pathway, HIF-1 pathway, and FoxO pathway
16	Perez-Duran et al. (2023) (38)	Observational retrospective cohort study	127 Caucasian CRC patients	The study did not provide any specific Vitamin D levels, however, the study evaluated the influence of 13 single nucleotide polymorphisms (SNPs) involved in the vitamin D metabolic pathway on CRC survival.	lymph node involvement, adjuvant chemotherapy, and no family history of CRC showed that the VDR ApaI (*p* = 0.036), CYP24A1 rs6068816 (*p* < 0.001), and GC rs7041 (*p* = 0.006) were associated with OS in patients diagnosed with CRC, and CYP24A1 rs6068816 (*p* < 0.001) was associated with PFS adjusted for metastasis, age of diagnosis, stage (IIIB, IV or IVB), ECOG score (2, 5, 6), lymph node involvement, adjuvant chemotherapy, and no primary tumor resection	SNPs mentioned above may have a key role as prognostic biomarkers of CRC.
17	Kim et al. (2021) (41)	Prospective cohort study	111 incident cases of early-onset CRC	400 IU/day increase	Higher total vitamin D intake was significantly associated with a reduced risk of early-onset CRC. The inverse association was significant and appeared more evident for dietary sources of vitamin D	In a cohort of younger women, higher total vitamin D intake was associated with decreased risks of early-onset CRC and precursors.

Two included articles—Reference (3) (animal study) and Reference (4) (commentary)—were retained despite not meeting inclusion criteria due to their conceptual and mechanistic relevance. Additionally, eight AI-related papers were removed after a corresponding section was excluded from the manuscript during revision.

**Figure 1:**
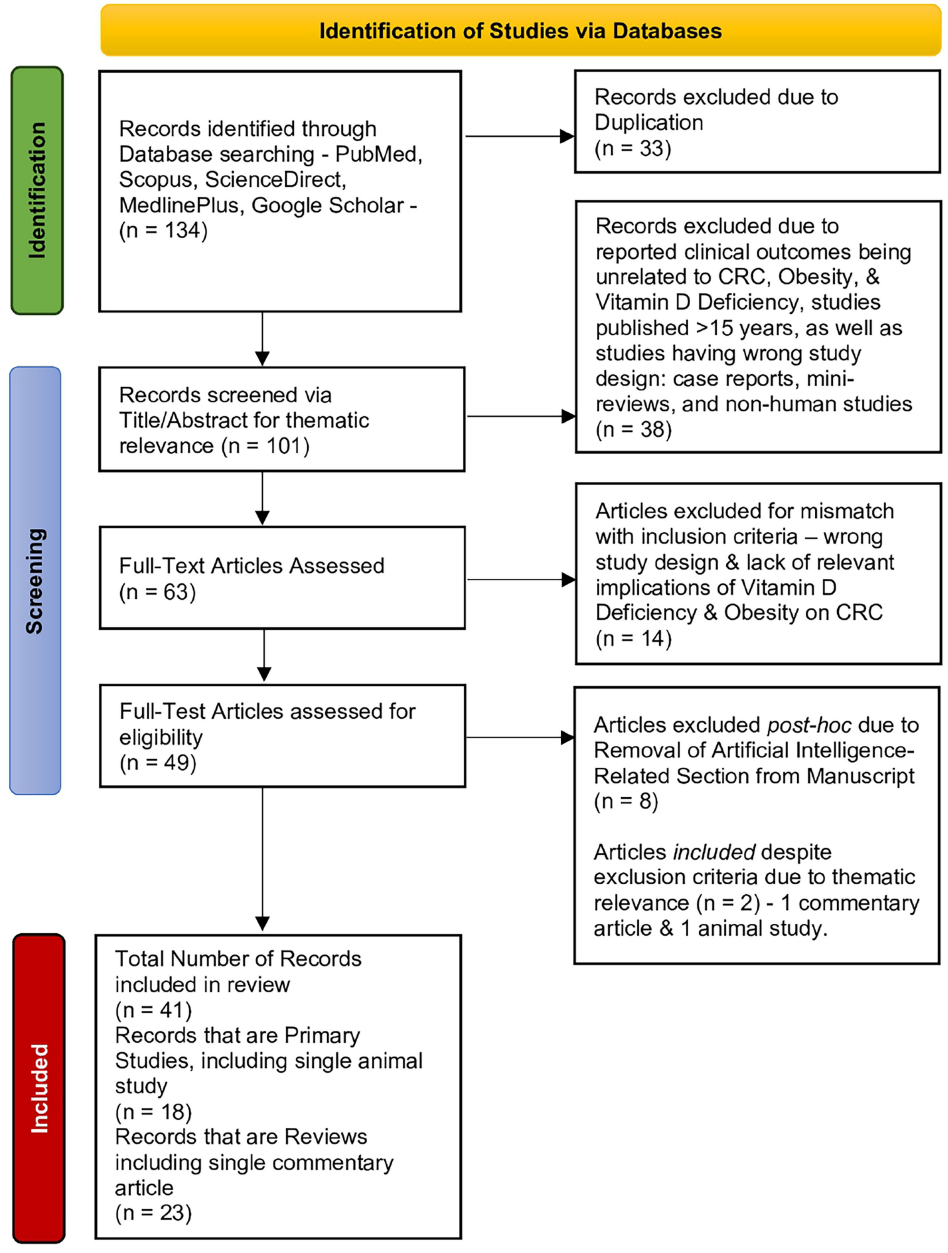
PRISMA flowchart illustrating the literature screening and inclusion process for studies addressing the interrelations among vitamin D deficiency, colorectal cancer outcomes, and obesity. Out of 134 initial records, 41 were included in the final review (18 primary studies and 23 reviews).

The selection process is illustrated in a PRISMA-style flow diagram to enhance transparency, even though the review did not follow a formal systematic protocol (Figure 1).

## 3 Mechanisms of vitamin D in colorectal carcinogenesis and progression

**Figure 2:**
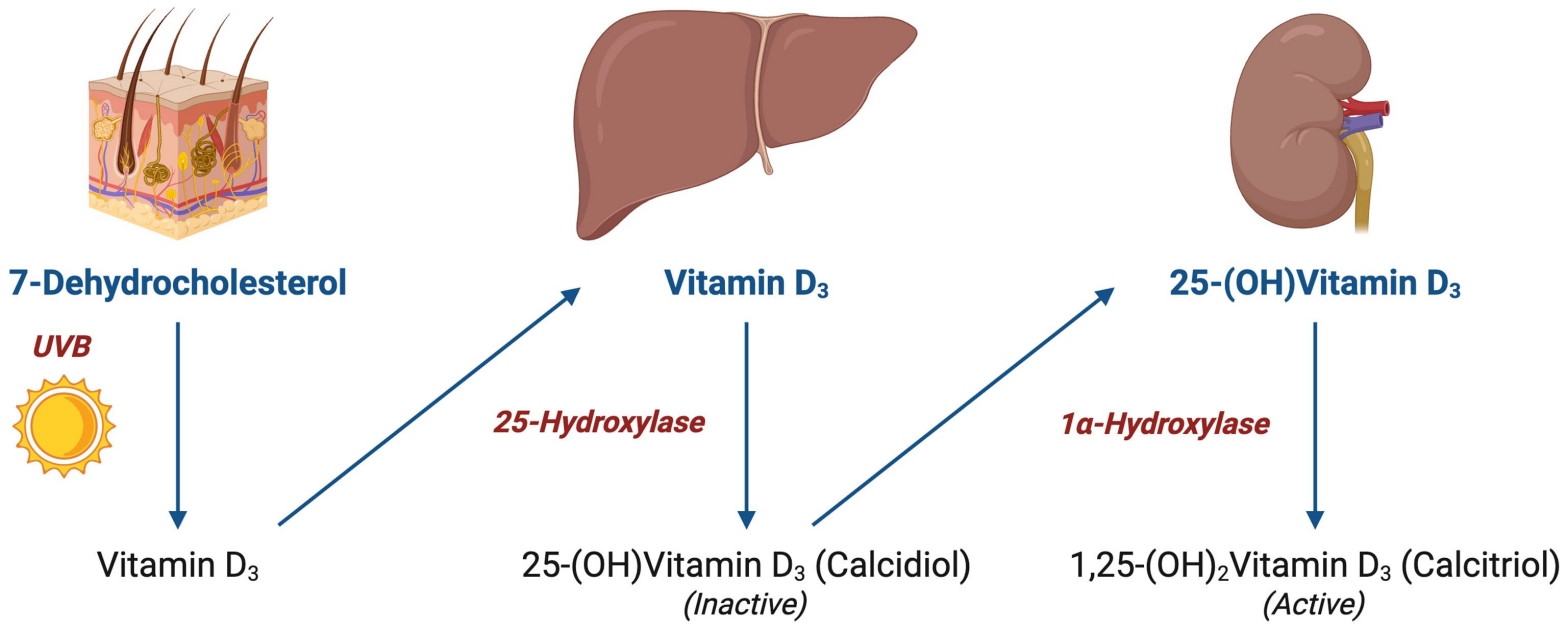
Vitamin D hydroxylation and conversion: illustration of vitamin D hydroxylation pathway, starting from 7-dehydrocholesterol in the skin to the formation of active 1,25-(OH)₂Vitamin D₃ via hepatic and renal conversion.

**Figure 3:**
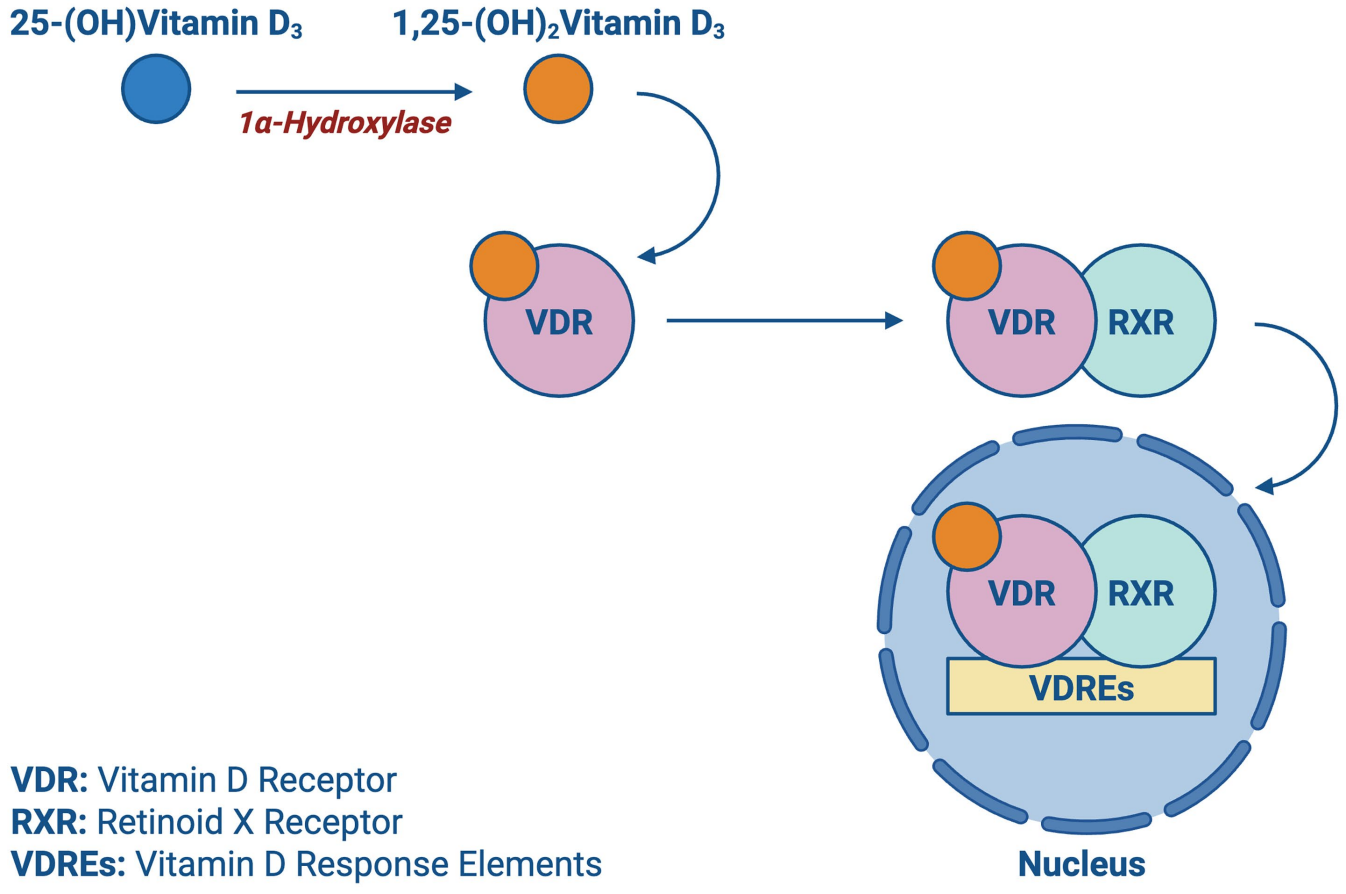
Vitamin D transcription: a schematic showing the activation of vitamin D receptor (VDR) by calcitriol and its subsequent nuclear translocation and interaction with vitamin D response elements (VDREs).

Upon entering the bloodstream, vitamin D is hydroxylated in the liver into 25-hydroxyvitamin D (calcidiol), the primary circulating form of vitamin D. This process is initiated primarily by cholecalciferol (vitamin D₃), which is generated in the skin upon exposure to UVB radiation. As shown in Figure 2, the 1α-hydroxylase enzyme in the kidney is responsible for further hydroxylation of 25-(OH) D_3_ into its active form 1,25-(OH)_2_D_3_ (calcitriol), which exerts its effects by binding to vitamin D receptors (VDR). As depicted in Figure 3, the VDR induces heterodimer complex formation with retinoid X receptor (RXR), the complex subsequently binds to vitamin D response elements (VDREs) in the promoter regions of target genes, regulating its transcription (5).

**Figure 4:**
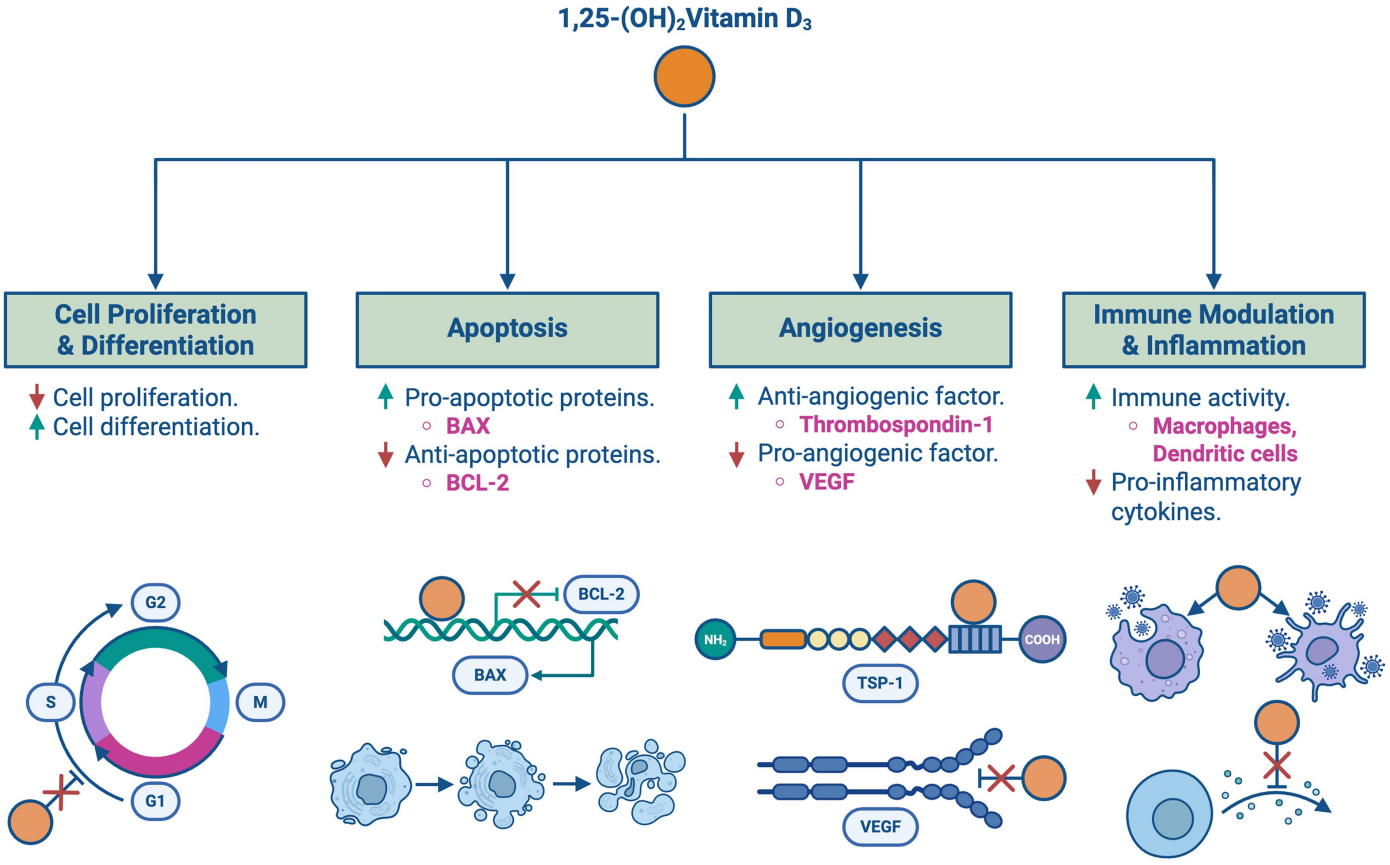
Vitamin D’s roles in cancer regulation: an overview of the key roles of 1,25-(OH)₂Vitamin D₃ in cancer regulation, including effects on cell proliferation, apoptosis, angiogenesis, and immune modulation. **(A)** Inhibits cell proliferation and promotes differentiation via regulation of cyclins and CDK inhibitors. **(B)** Induces apoptosis by upregulating pro-apoptotic factors such as BAX and downregulating anti-apoptotic proteins like BCL-2. **(C)** Inhibits angiogenesis by downregulating VEGF and increasing anti-angiogenic factors such as thrombospondin-1. **(D)** Modulates immune responses by enhancing macrophage and dendritic cell activity while suppressing pro-inflammatory cytokines.

As shown in Figure 4, vitamin D inhibits cell proliferation while promoting differentiation as it influences the gene expression of various proteins involved in cell cycle regulation, such as cyclins and cyclin-dependent kinases, and CDK inhibitors, causing cell cycle arrest in the G1/S phase. Furthermore, vitamin D has been shown to induce apoptotic responses in cancer through the upregulation of pro-apoptotic factors like BAX and the downregulation of anti-apoptotic proteins such as BCL-2 (5).

Angiogenesis is an essential process for tumor growth and spread. Vitamin D downregulates the expression of pro-angiogenic factors such as vascular endothelial growth factor (VEGF) and upregulates the expression of anti-angiogenic factors including thrombospondin-1, a tissue inhibitor of metalloproteinases. Additionally, vitamin D is involved in modulating immune responses and inflammation, which is crucial in controlling cancer progression through upregulating the activity of immune cells such as macrophages and dendritic cells and suppressing pro-inflammatory cytokines (5).

The pathophysiological role of vitamin D in colorectal cancer (CRC) is supported by substantial evidence. Several epidemiological studies have demonstrated that lower serum levels of 25(OH)D₃ are associated with an increased risk of CRC, with a reported hazard ratio (HR) of 1.62 (95% CI: 1.04–2.53) (6). This inverse relationship is thought to be mediated through the mechanisms of cell differentiation, apoptosis, and proliferation.

Increased risk of colorectal neoplasms, including adenomatous polyps, has been linked to vitamin D deficiency. A cross-sectional study conducted by Zamora-Ros et al. (2023) highlighted that low serum 25(OH)D₃ levels were significantly associated with the presence of colorectal polyps (CRPs), particularly in individuals aged 50 to 64 years (OR = 1.81, 95% CI: 1.12–2.92, *p* = 0.016) (7). Vitamin D deficiency, combined with metabolic disorders such as hyperglycemia and elevated triglycerides, further increases the risk of developing colorectal polyps (CRPs). The overall odds ratio (OR) of metabolic syndrome (MetS) for CRPs was 2.50 (95% confidence interval [CI] = 1.95–3.21) (7).

Genetic variations in the VDR gene have been associated with a higher risk of developing CRC as well. A study conducted by Lv et al. (2021) demonstrated that polymorphisms in the VDR gene affect its functional ability to regulate target genes involved in cell growth and differentiation, as certain VDR gene polymorphisms were associated with an increased susceptibility to CRC, emphasizing the significance of vitamin D signaling pathway in colorectal carcinogenesis (8).

Emerging evidence also suggests that vitamin D may exert its protective effects through modulation of the gut microbiome. Gut microbiotas play a critical role in colorectal homeostasis by regulating immune tolerance, epithelial integrity, and inflammatory signaling functions that overlap with vitamin D’s known actions. Vitamin D influences microbiota composition via its receptor (VDR), bolstering the growth of beneficial microbial taxa and suppressing dysbiotic profiles associated with tumorigenesis. These effects are particularly relevant in CRC, where dysbiosis contributes to barrier dysfunction and chronic mucosal inflammation, thereby facilitating carcinogenic progression (4).

Moreover, vitamin D enhances the structural integrity of the gut barrier by upregulating the expression of tight junction proteins such as claudins and occludins, thus reducing intestinal permeability and bacterial translocation. It also regulates the local immune environment by modulating cytokine production and promoting regulatory T cell (Treg) responses, which limit chronic inflammation in the colonic mucosa. This interplay between vitamin D, microbiota, and mucosal immunity has shown promising results in preclinical and translational studies, highlighting a potential adjunctive role of vitamin D in microbiota-targeted cancer therapy (9).

## 4 Serum vitamin D status and prognostic outcomes in colorectal cancer

Vitamin D deficiency may influence the progression and prognosis of CRC as adequate serum 25-(OH)D_3_ levels have been associated with improved outcomes. For instance, in a prospective cohort study by Wesselink et al. (2020), CRC patients with both sufficient serum 25-(OH)D_3_ concentrations (≥50 nmol/L) and high dietary magnesium intake (≥322 mg/d or ≥383 mg/d depending on cohort) had a 47% lower risk of all-cause mortality (Hazard Ratio: 0.53; 95% CI: 0.31–0.89) compared to those with deficient vitamin D and low magnesium intake. Furthermore, within the subgroup of patients with sufficient vitamin D, high magnesium intake was associated with a 57% reduction in mortality (HR: 0.43; 95% CI: 0.25–0.76). These findings suggest two key points: reduced vitamin D concentrations seem to be associated with a higher risk of all-cause mortality in CRC patients, and the data points at a possible synergistic role between Vitamin D status and magnesium intake in CRC prognosis (given that magnesium is essential in the conversion of 25-(OH)D_3_ to the active form of vitamin D).

These findings align with a large-scale international pooling project by Weinstein et al. (2018), which analyzed data from 17 cohorts that tested 5,706 CRC case participants and 7,107 control participants with a wide range of circulating 25-(OH)D_3_ concentrations and confirmed an inverse association between vitamin D levels and CRC risk, as individuals that had 25-(OH)D_3_ deficiency had a 31% higher risk of CRC, particularly among women and those with initially low vitamin D levels (10). This extensive study underscores the importance of maintaining sufficient vitamin D levels as a potential preventive measure against CRC. Other studies, such as the population-based study by Keum et al. (2019), further demonstrate that individuals with vitamin D deficiency have a significantly increased risk of developing CRC, even after accounting for various confounders such as age, sex, lifestyle, and other health conditions. Based on this, the association between low vitamin D levels and increased CRC risk is significant, therefore suggesting that vitamin D deficiency is an independent risk factor for CRC (11).

Geographic location, skin pigmentation, and dietary habits are also significant determinants of vitamin D status. Individuals residing at higher altitudes with less sunlight exposure are more likely to experience vitamin D deficiency due to reduced opportunities for skin synthesis of vitamin D. Similarly, people with darker skin pigmentation have higher melanin levels, which can reduce the skin’s ability to produce vitamin D in response to sunlight. Dietary habits also play a crucial role, as diets low in vitamin D-rich foods, such as fatty fish, fortified dairy products, and egg yolks can contribute to deficiency (12).

Collectively, these studies provide compelling evidence for the multifaceted role of vitamin D in reducing the risk of CRC while also highlighting the importance of environmental, genetic, and lifestyle factors when assessing vitamin D status.

## 5 Obesity, vitamin D, and colorectal cancer prognosis

**Figure 5:**
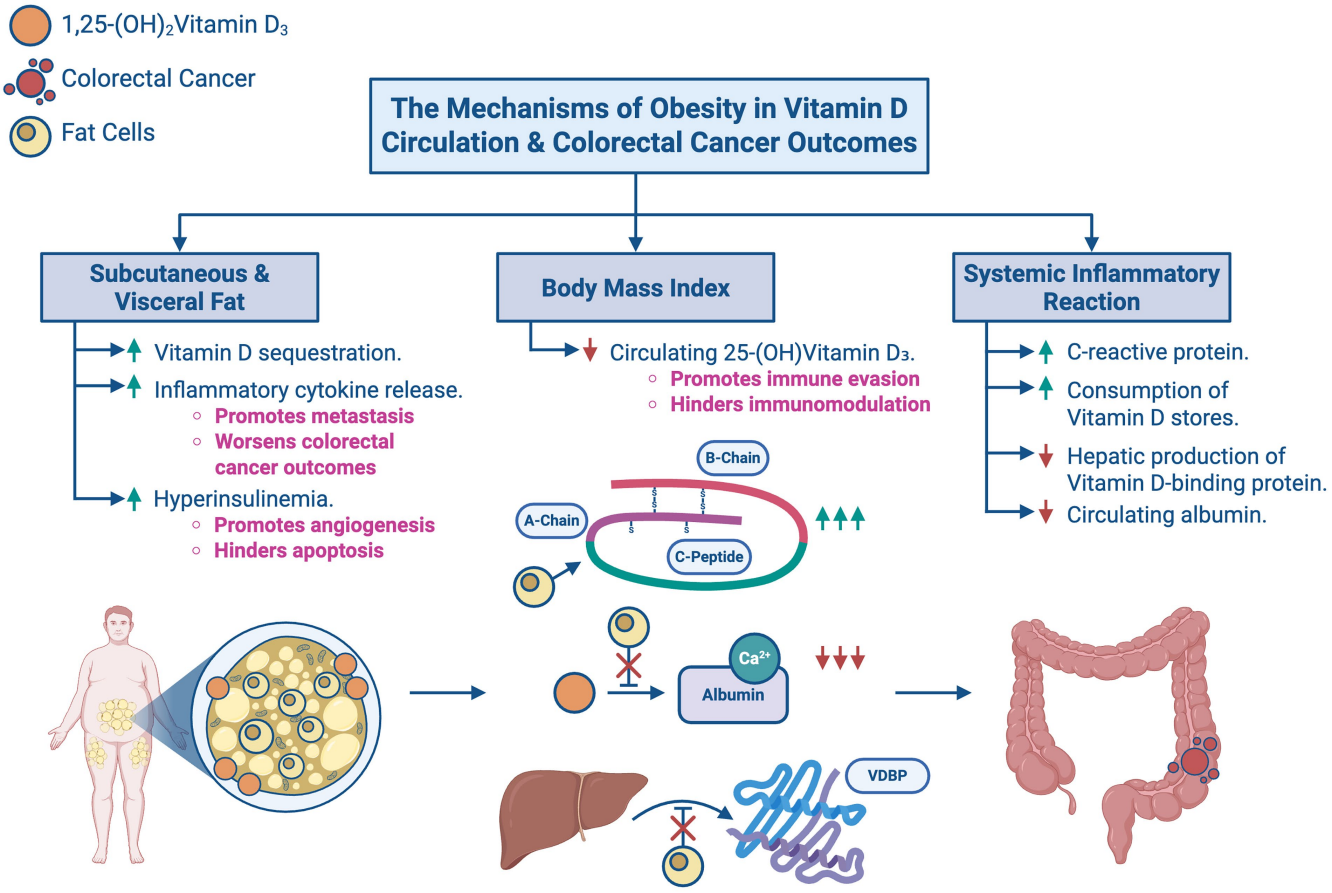
The mechanisms of obesity in Vitamin D circulation and colorectal cancer outcomes: an overview of how obesity modulates Vitamin D levels and influences colorectal cancer outcomes. **(A)** Subcutaneous and visceral fat promotes Vitamin D sequestration, inflammatory cytokine release, and hyperinsulinemia. **(B)** Increased body mass index reduces circulating 25-(OH)Vitamin D3, thereby promoting immune evasion and hindering immunomodulation. **(C)** Systemic inflammatory reaction causes an increase in CRP and consumes Vitamin D stores while reducing hepatic production of Vitamin D-binding protein (VDBP) and circulating albumin.

As shown in Figure 5, obesity has been shown to affect serum 25-(OH)D_3_ levels and CRC prognosis, with some studies reporting a direct relationship between the three. Obesity affects vitamin D metabolism by increasing its storage in subcutaneous fat, leading to circulating deficiency. One case–control study by Väyrynen, J. P. et al. (2016) reviewing 25-(OH)D_3_ deficiency and prognosis in CRC patients concluded CRC patients with BMI > 30 have serum 25-(OH)D_3_ deficiency compared to those with a BMI ≤ 30. This implies that patients with a greater BMI are likely to have lower serum 25-(OH)D_3_ levels (13).

However, it is worthwhile to note that the same case–control study hinted that the effects of BMI on serum 25-(OH)D_3_ levels may have been confounded by systemic inflammatory responses. These responses control metastasis and thereby influence the association between 25-(OH)D_3_ concentrations and CRC via the immunomodulatory and immunosuppressive functions of vitamin D19 and proinflammatory cytokines released due to cancer progression. These suppress the hepatic production of vitamin D carrier proteins resulting in the redistribution or consumption of vitamin D storages. In other words, high systemic inflammatory responses to CRC do not only reduce serum albumin levels and increase serum CRP levels, but they can also potentiate vitamin D deficiency, eliciting a worse prognosis. Furthermore, a multivariate analysis by Conway and McMillan perceived that systemic inflammatory responses had a higher association with low serum 25-(OH)D_3_ levels than high BMI (13).

A meta-analysis by Pereira-Santos, M. et al. (2015) focused on the relationship between 25-(OH)D_3_ deficiency and body measurements and discovered that obese individuals have prevalence ratio (PR) of 35% for vitamin D deficiency compared to normal-weight individuals. In the subgroup analysis, obese children and adolescents had a 37% PR, while obese adults had a 33% PR of vitamin D deficiency (14). These findings conclude that obesity is associated with a higher prevalence of vitamin D deficiency, therefore implying that obesity has an indirect effect on CRC prevalence.

Regarding the combined impact of BMI and Obesity, a study by Budny A. et al. (2019) found that increased visceral adipose tissue levels are associated with hyperinsulinemia, another risk factor for CRC (15). Further emphasizing the effects of hyperinsulinemia, a study by Zhang A. M. Y. et al. (2021) found that the percentage of cancer cases associated with obesity and diabetes increased by 20 and 30%, respectively, between 1980 and 2002. Notably, the same study stated that while obesity and diabetes contribute to hyperinsulinemia, hyperinsulinemia itself can also act as an independent factor for cancer risk. These findings underscore the complexity of the relationship and highlight the importance of identifying the underlying mechanisms that link cancer with obesity and diabetes (16).

Further investigating the potential benefits of weight management and vitamin D supplementation in improving CRC outcomes, a study by Wesselink, E. et al. (2020) explaining vitamin D’s role in CRC outcomes, suggested improved survival rates with increased 25-(OH)D_3_ levels that can be adjusted through dietary and lifestyle modifications, including moderate-to-vigorous outdoor activity during solar noon, vitamin D supplementation, and calcium intake. However, 25-(OH)D_3_ levels can change over time due to dietary adjustments after diagnosis or treatment. Median 25-(OH)D_3_ levels decreased over time in CRC patients undergoing surgery or chemotherapy. To counter that, vitamin D supplements could be administered, whereas supplementation at 6 months post-diagnosis results in a 4 nmol/L less reduction in vitamin D levels compared to non-users. Alcohol consumption should be taken into consideration as a confounder as it may also affect serum levels (17).

In addition, a meta-analysis by Boughanem, H. et al. (2021) evaluated 47,540 cases and 70,567 controls in case–control studies and discovered a 25% reduced risk of CRC when comparing the highest to the lowest dietary vitamin D consumption. The meta-analysis also examined 14,676 CRC-incident cases in prospective cohorts from 16 countries, but they did not show any significant associations thus further analytic confirmation is required (18).

## 6 Therapeutic implications of vitamin D supplementation

Several randomized controlled trials (RCTs) that examined the effectiveness of vitamin D supplementation in CRC exhibited no significant reduction of ≥15% in relative risk of cancer or ≥10% in cancer mortality with the provision of vitamin D supplementation. These analyses also did not differentiate between the effects of calcium and vitamin D on CRC prognosis (19).

Furthermore, the Keum et al. (2019) study, which evaluated RCTs on vitamin D supplementation and total cancer rates, supports the prior meta-analyses by Beatriz, G. et al. However, this study has limitations—such as low dosing, mixed populations, and limited follow-up (20). Therefore, instead of relying solely on these secondary analyses, we synthesized CRC-specific data: Vaughan-Shaw et al. (2020) observed a 30% improvement in survival, while Bellerba et al. (2022) found reduced recurrence with daily supplementation. These findings suggest potential therapeutic benefit in CRC when higher or consistent dosing is applied.

Conversely, a meta-analysis by Keum et al. (2014) reported no statistically significant association between vitamin D supplementation and overall cancer incidence. Supporting this, a 2022 meta-analysis of RCTs by Emmanouilidou G. et al. found that vitamin D supplementation did not appear to play a chemopreventive role in colorectal neoplasms. Nonetheless, both reviews acknowledged the limitations in existing studies—particularly the use of low vitamin D doses (≤1,100 IU/day) and small sample sizes—highlighting the need for further investigation in this area (19). Interestingly, while these studies failed to establish a role for vitamin D in reducing cancer incidence, some observed modest but notable reductions in cancer-related mortality (see Table 2 for summarized findings from major studies). For instance, more recent data suggest that daily vitamin D supplementation may reduce cancer mortality by approximately 13%, with a relative risk (RR) of 0.87 (95% CI: 0.78–0.96), although this effect did not vary significantly across different subgroups (20). Moreover, the initial Keum meta-analysis also reported a 7% reduction in overall mortality, of which one-third of deaths were attributed to cancer. While these findings are not definitive, they suggest a potential survival benefit, particularly when consistent, higher-dose regimens are utilized—underscoring the necessity for larger, well-designed trials to clarify vitamin D’s therapeutic role in CRC prevention and prognosis (20).

**Table 2:** Summary of statistical findings on the impact of Vitamin D supplementation on colorectal cancer prognosis and therapeutic dosages of Vitamin D.

Authors and Year	Vitamin D supplementation dosage or serum level	Reported risk reduction or other statistical findings/key outcomes related to colorectal cancer
Hatim Boughanem et al. (2021) (18)	No exact mention about the Vitamin D serum level or dosage	A significant 25% lower risk was reported comparing the highest vs. lowest dietary Vitamin D consumption & CRC risk
Federica Bellerba et al. (2022) (26)	25(OH)D ≥ 30 ng/mL	12% Reduction in CRC recurrence with vitamin D supplementation
Liam Sutherland et al. (25)	600 IU/day	22% Reduction in high-risk adenomatous polyps with ≥600 IU/day vitamin D intake
Fuchs et al. (2017) (27)	27.6 ng/mL	25% Improvement in disease-free survival in patients with high vitamin D levels
Evertine Wesselink et al. (2020) (28)	≥50 nmol/L	57% Reduction in all-cause mortality with sufficient vitamin D (≥50 nmol/L) and magnesium intake
David Corley Gibbs et al. (2023) (24)	1,000 IU/day	Recommended daily dose of vitamin D for supplementation
Keum et al. (2019) (20)	2000 IU/day to 100,000 IU/month	High doses of vitamin D used in large RCTs

Another study assessing the effect of vitamin D supplementation on survival rates showed a 30% reduction in adverse CRC outcomes (21). Additionally, a systematic review by Ajebli et al. found that while vitamin D alone may not prevent precancerous growth, its combination with omega-3 fatty acids not only improved inflammatory markers but also showed potential, indirect prognostic benefits by reducing tumor-promoting inflammation and enhancing immune response (22).

One study tested the effect of a personalized vitamin D₃ loading dose to optimize 25-(OH)D_3_ levels. While the intervention safely increased serum levels with low cost and minimal adverse effects, the reported 12% reduction in CRC mortality was based on modeled projections rather than direct clinical outcome data (23). Thus, while promising, the mortality impact remains suggestive and warrants further validation.

Vitamin D doses are usually recommended within the range of 600 to 1,000 IU per day. The typical clinical approach is a daily combination of 1,000 IU of vitamin D_3_ with 1,200 mg of calcium, which has been shown to reduce adenoma recurrence, especially in patients with a genetic predisposition to low vitamin D levels (24). Gigic et al. (2022) emphasized the importance of consistency in taking supplements during the first 6 months following diagnoses when patients are undergoing chemotherapy, which may decrease levels of vitamin D. It is taken orally specifically through cholecalciferol (vitamin D_3_) due to its easy absorption and high bioavailability (17). In areas with limited sunlight such as high-latitude regions, McGregor et al. (2020) suggested that a daily intake of ≥600 IU can successfully cut down on high-risk adenomatous polyps by 22% (25). It is taken orally specifically through cholecalciferol (vitamin D_3_) due to its easy absorption and high bioavailability (17). In areas with limited sunlight such as high-latitude regions, McGregor et al. (2020) suggested that a daily intake of ≥600 IU can successfully cut down on high-risk adenomatous polyps by 22% (25). These levels are maintained using the daily dosing protocol as confirmed by blood test results, checking optimal serum concentration of 25-(OH)D_3_, and patient-specific adjustments.

Building upon these dosing strategies, recent high-quality evidence reinforces the therapeutic benefits of maintaining optimal vitamin D levels in CRC patients. The Bellerba et al. (2022) study demonstrated that patients receiving vitamin D supplementation exhibited a 12% reduced risk of recurrence compared to controls, with a hazard ratio (HR) of 0.88; 95% CI: 0.78–0.99 (26). This protective effect aligns with the findings of Fuchs et al. (2017), who reported a 25% improvement in disease-free survival (HR: 0.75; 95% CI: 0.60–0.93) among CRC patients with higher predicted vitamin D status (27). This is also complemented by the results from Wesselink et al. (2020) study discussed earlier that mentioned the 57% reduction in all-cause mortality derived from the COLON study when there are sufficient serum 25-(OH)D_3_ levels (≥50 nmol/L) supported by adequate magnesium intake (28). In parallel, emerging research on genetic determinants such as VDR polymorphisms, as reviewed by Pereira et al. (2024), suggests that individual genomic profiles may further modulate responsiveness to vitamin D therapy. Together, these findings not only corroborate the physiological rationale for supplementation but also highlight its real-world clinical impact across multiple endpoints in CRC care (29).

## 7 Genetic predispositions and the interaction with vitamin D in colorectal Cancer

**Figure 6:**
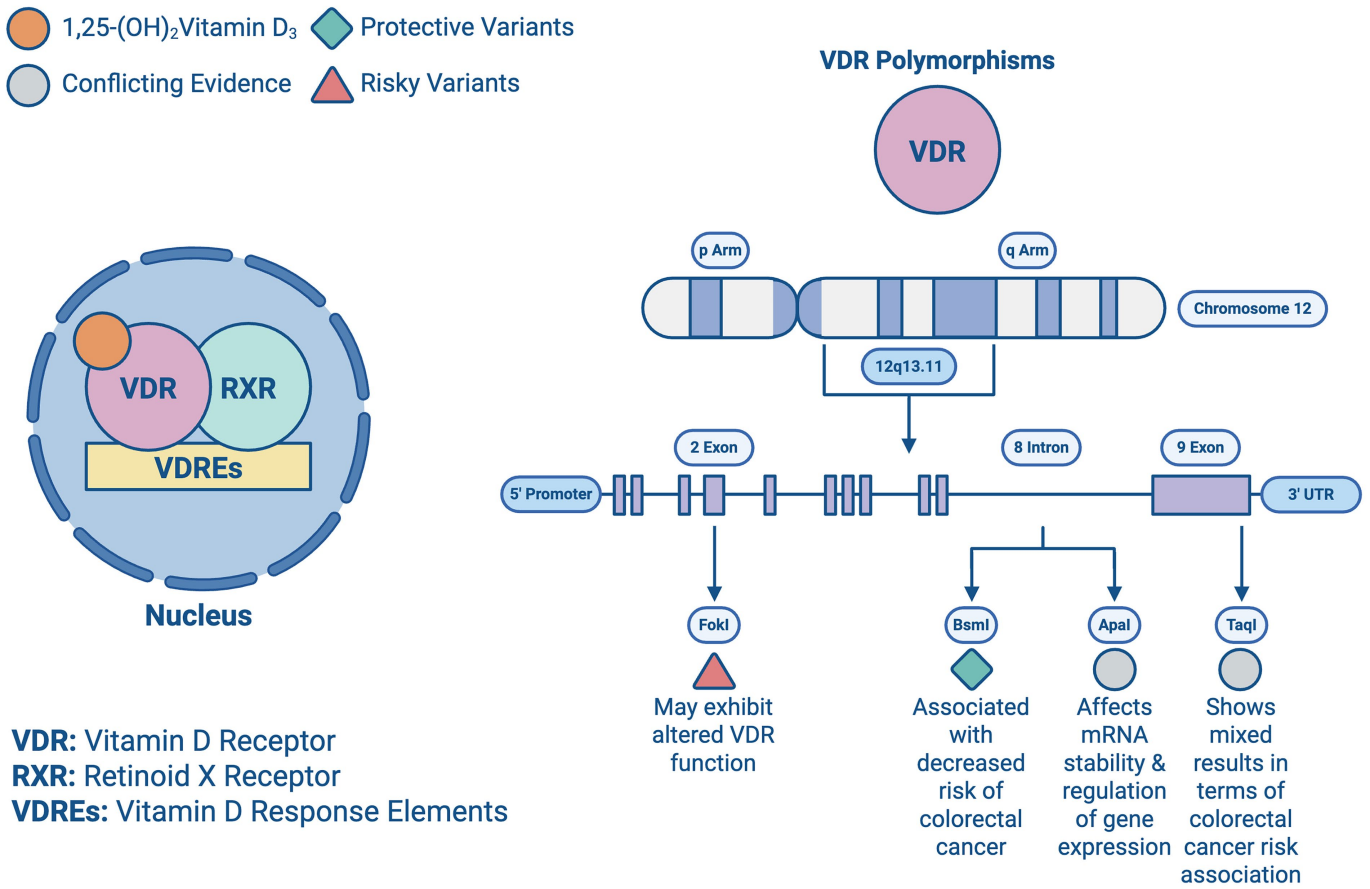
Vitamin D receptor polymorphisms: illustration of the Vitamin D transcription from a previous figure and all the vitamin D receptors (VDR) present in chromosome 12 and their beneficial or risky outcomes.

The expression of VDR in epithelial and stromal colon cells plays a crucial role in tumor progression. While some CRC cell lines retain VDR expression, others lose it, becoming resistant to the antitumor effects of 1,25-(OH)_2_D_3_. VDR expression typically increases in precancerous lesions but decreases in later, advanced stages, limiting the potential effectiveness of VDR agonists (8, 29, 30). Key polymorphisms such as ApaI, TaqI, BsmI, and FokI, which are essential for vitamin D’s biological functions, affect mRNA stability and gene expression regulation, with their prognostic associations summarized in Figure 6 and Table 2. These variants can disrupt the vitamin D pathway, potentially impacting CRC development (3, 31, 32).

Heritable factors account for about 35% of the disease risk, despite less than 5% of cases being directly attributed to genetic predisposition. Variations in genes like VDR are important, as they modulate both the risk and progression of CRC. The BsmI polymorphism, for instance, has been associated with a decreased risk of CRC, particularly among Caucasians, while the TaqI polymorphism has shown mixed results in terms of risk association. The complex interplay between these genetic factors and vitamin D levels suggests a multifaceted influence on CRC prognosis (33–35).

Studies highlight the interaction between genetic predispositions and vitamin D levels in CRC. High VDR expression in stromal fibroblasts generally correlates with better outcomes, suggesting that even patients with low VDR-expressing tumor cells might benefit from VDR agonists if their stromal cells are adequately expressing VDR. Additionally, microRNAs such as miR-27b and miR-372/373 can downregulate VDR, influencing CRC resistance to vitamin D (36, 37). Studies investigating gene-vitamin D interactions have provided insights into how genetic predispositions modulate the effects of vitamin D on CRC. Meta-analyses have demonstrated that certain VDR polymorphisms, such as BsmI and Cdx-2, correspond with an altered CRC risk, like the BsmI variant showing protective effects in specific populations. These interactions highlight the importance of considering genetic background when assessing vitamin D’s role in CRC progression and prognosis (38–40).

Based on this information, the potential for personalized medicine utilizing genetic testing to tailor vitamin D supplementation in CRC patients, enhance therapeutic efficacy, and improve patient outcomes seems promising. Recent studies have shown that specific polymorphisms and mutations in the VDR gene can significantly influence the effectiveness of vitamin D treatment. Individuals with the FokI polymorphism may exhibit altered VDR function, necessitating personalized dosing strategies to achieve optimal therapeutic levels (40). Similarly, while the BsmI and TaqI polymorphisms have been widely studied, current evidence shows inconsistent associations with treatment outcomes; however, their potential role in modulating vitamin D responsiveness in specific subpopulations (like IBD patients) continues to be explored (3). Understanding a patient’s VDR genotype can help healthcare providers design customized supplementation plans that maximize the antitumor effects of vitamin D, potentially reducing CRC risk and improving prognosis (32, 33). Additionally, the integration of microRNA profiling with genetic testing could further refine personalized treatment approaches. MicroRNAs such as miR-27b and miR-372/373 could serve as biomarkers for resistance to vitamin D therapy, enabling clinicians to identify patients who would benefit most from alternative or adjunctive treatments (34). This precision medicine approach aligns with the broader trends in oncology, aiming to tailor interventions based on individual genetic and molecular profiles.

The interplay between genetic predispositions and vitamin D in CRC is complex and ever-changing. Understanding the specific genetic variants affecting vitamin D metabolism, alongside broader genetic susceptibility to CRC, is crucial for developing targeted interventions. Future research should focus on large-scale, multi-ethnic studies to validate these findings and optimize personalized vitamin D supplementation strategies in clinical practice (35). By integrating genetic information with nutritional interventions, there is potential to significantly impact the management and prognosis of CRC (40) (Table 3).

**Table 3:** Genetic polymorphisms and their associations with colorectal cancer risk and Vitamin D responsiveness.

Polymorphism name	Gene/miRNA	Effect on CRC or Vitamin D pathway	Statistical association	Reference(s)
BsmI (rs1544410)	VDR	Associated with reduced CRC risk, particularly in Caucasian populations	OR = 0.79; 95% CI = 0.65–0.96; *p* = 0.02	Yang et al. (2023) and Bai et al. (2012) (34, 36)
TaqI (rs731236)	VDR	No consistent association with CRC risk; varies across ethnicities	OR = 0.97; 95% CI = 0.87–1.08; *p* = 0.60 (NS)	Yang et al. (2023) and Bai et al. (2012) (34, 36)
ApaI (rs7975232)	VDR	Alters mRNA stability; may influence vitamin D transcriptional effects	No significant CRC association reported	Latacz et al. (2021) and Usategui-Martin et al. (2022) (8, 32)
FokI (rs2228570)	VDR	Modulates VDR protein activity and response to vitamin D; affects survival	Not significant for CRC risk (OR = 1.10; 95% CI = 0.95–1.29); impacts supplementation response	Bai et al. (2012), Usategui-Martin et al. (2022), and Perez-Duran et al. (2023) (36, 38)
Cdx2 (rs11568820)	VDR (promoter)	Affects transcription efficiency; relevant for survival and expression differences	No CRC risk statistics; prognostic marker in some populations	Perez-Duran et al. (2023)(38)
miR-27b, miR-372/373	microRNAs	Suppress post-transcriptional VDR expression; impact therapy resistance and vitamin D pathway	No ORs reported; supported by *in vitro* molecular data	Sluyter et al. (2021) (37)
rs4588	GC (VDBP)	Decreases bioavailable 25(OH)D; affects response to vitamin D supplementation	No CRC risk stats; associated with lower serum vitamin D levels	Rozmus et al. (2020) and Usategui-Martin et al. (2022) (32, 39)
rs7041	GC (VDBP)	TG genotype associated with CRC risk; alters vitamin D transport efficiency	Significant association with CRC risk (Chi^2^ *p* < 0.05 in Polish cohort)	Rozmus et al. (2020) (39)

## 8 Discussion

The impact of vitamin D supplementation on the prognosis of CRC presents a critical intersection of nutritional science and oncology, reflecting both the therapeutic potential and the complexities of clinical applications. This article consolidates the insights summarized from numerous studies, emphasizing the role of vitamin D in CRC prognosis and potential therapy.

Current literature consistently demonstrates an inverse relationship between serum vitamin D levels and the incidence of CRC. For instance, Peixoto and de Carvalho Oliveira (2022) highlighted that higher serum levels of vitamin D are associated with a reduced risk of CRC, aligning with the findings of Hernández-Alonso and Boughanem (2023), who confirmed that adequate vitamin D levels significantly lower CRC risk. These studies underscore the preventive role of vitamin D, suggesting that maintaining sufficient levels could potentially diminish the onset of CRC, particularly in high-risk populations.

However, the conversation shifts when considering the influence of vitamin D supplementation on CRC prognosis. While epidemiological evidence firmly supports the protective role of vitamin D, the impact of supplementation in influencing disease outcomes, particularly in those already diagnosed with CRC, remains less clear. Santorsola et al. (2024) noted low serum vitamin D levels pre-chemotherapy can negatively impact outcomes in metastatic CRC patients, implying that supplementation might improve prognosis. Yet, this remains a complex area of study, where the interplay of dosage, timing, pathological staging, and individual patient factors, such as baseline vitamin D levels, genetic predispositions, and social factors, requires further exploration.

Clinical trials investigating vitamin D supplementation have yielded mixed results. Vaughan-Shaw et al. (2020) demonstrated a 30% reduction in adverse outcomes among CRC patients receiving vitamin D supplementation. Despite this promising figure, the variation in study designs, dosage regimens, clinical staging, and patient populations across different trials complicates the generalizability of these findings. For instance, the study by Ng et al. (2019) involving high-dose vitamin D_3_ supplementation in metastatic CRC patients showed a modest improvement in progression-free survival. However, the overall survival benefit was not statistically significant. These outcomes highlight the need for more standardized, large-scale trials accounting for confounding variables to clarify the potential therapeutic benefits of vitamin D in CRC management.

Moreover, it is important to note that most RCTs focus exclusively on oral supplementation without addressing UV exposure, the primary physiological source of vitamin D. Given that supplementation may only marginally impact serum calcitriol levels, future research should also consider therapeutic sunlight exposure, particularly in regions or patient populations where deficiency is driven by limited UV access rather than dietary insufficiency.

Also, in the context of inter-individual variability, the role of genetic factors cannot be overlooked. Genetic polymorphisms in the VDR gene, as discussed by Lv et al. (2021), can significantly influence the efficacy of vitamin D supplementation. For example, certain VDR gene variants may enhance or diminish the body’s response to vitamin D, potentially altering the therapeutic outcomes in CRC patients. This genetic variability underscores the importance of personalized medicine approaches in optimizing vitamin D therapy, where genetic testing could guide individualized supplementation strategies.

Furthermore, the relationship between obesity, vitamin D deficiency, and CRC prognosis introduces additional complexity. Obesity, a known risk factor for CRC, is often associated with lower serum vitamin D levels due to sequestration within adipose tissue. Väyrynen et al. (2016) indicated that CRC patients with a higher BMI tend to have lower serum vitamin D levels, which may partially explain the poorer outcomes observed in this patient group. This interplay suggests that concurrently addressing obesity and vitamin D deficiency could be a critical component of CRC management.

In conclusion, while the protective role of vitamin D against CRC development is well-supported, its impact on CRC prognosis through supplementation remains a controversial and evolving field. Current literature suggests potential benefits, particularly in specific subgroups of patients, but also highlights the deficits in current trials and the need for more targeted research. Future studies should focus on elucidating the optimal dosing strategies, understanding the role of genetic factors, and exploring the combined effects of vitamin D supplementation and weight management in improving CRC outcomes. Through these avenues, vitamin D may become a cornerstone of a more personalized and effective approach to CRC treatment and potential prevention.

The relationship between obesity, vitamin D deficiency, and CRC prognosis introduces additional complexity. Obesity, a known risk factor for CRC, is associated with lower serum 25-(OH) D_3_ levels due to sequestration in adipose tissue and may confound the impact of supplementation. Väyrynen et al. (2016) showed that higher BMI in CRC patients correlated with lower vitamin D levels, possibly contributing to poorer outcomes. However, it is critical to recognize that 80–90% of circulating calcitriol is derived from UV exposure, not diet. Thus, dietary supplementation—though practical—may only modestly influence systemic levels in CRC patients, many of whom also face reduced intake and absorption due to illness or treatment. This underscores a neglected therapeutic consideration: controlled sunlight or UV exposure may offer greater physiological impact than oral supplementation alone. Despite observational links between vitamin D and CRC prognosis, there is significant heterogeneity in trial results. For example, closer reading of the COLON study shows that survival benefits were notably influenced by magnesium intake, and not solely by vitamin D status (Wessellink et. Al - 2020). Therefore, while the literature supports a role for vitamin D, publication bias and selective reporting of positive results remain concerns. A more balanced synthesis—considering confounders, non-supplemental sources of vitamin D, and methodological quality—is essential to clarify vitamin D’s true prognostic value in CRC (Tables 4, 5).

**Table 4:** Comparison of colorectal cancer outcomes across Vitamin D serum levels.

Study	Vitamin D level (25-(OH)D_3_)	CRC outcome studied	Finding	HR/RR/OR	Extra notes
Wesselink et al. (2020) (28)	≥50 nmol/L	All-cause mortality in CRC	Lower vitamin D correlated with increased mortality	HR: 0.53 (CI: 0.31–0.89)	Synergistic with high magnesium intake
Weinstein et al. (2018) (10)	Varied (wide range)	CRC Incidence	Low 25-(OH)D_3_ levels associated with higher CRC risk	+31% CRC risk	Largest pooled analysis (17 cohorts)
Keum et al. (2019) (11)	Deficient vs. sufficient	CRC Risk	Vitamin D deficiency increased CRC risk	Not specified	Controlled for age, sex, and confounders
Väyrynen et al. (2016) (13)	Low in BMI > 30 group	CRC Prognosis	Obese patients had lower levels and worse outcomes	Indirect	Confounded by inflammation
Vaughan-Shaw et al. (2020) (21)	Supplemented vs. unsupplemented	CRC-specific survival	Vitamin D supplementation improved survival	~30% better outcome	High-dose daily dosing
Fuchs et al. (2017) (27)	Predicted high serum level	Disease-free survival (DFS)	Higher vitamin D predicted better DFS	HR: 0.75 (CI: 0.60–0.93)	Based on predictive models
McGregor et al. (2020) (25)	≥600 IU/day (oral intake)	High-risk adenomatous polyps	Lower incidence of adenomas	22% reduction	Latitude and sunlight important factors

**Table 5:** Summary of statistical associations between Vitamin D supplementation, sunlight exposure, and CRC prognosis.

Factor	Effect on CRC prognosis	Statistic/Finding	Supporting studies
Oral Vitamin D supplementation	Improved survival, reduced recurrence in CRC	12% reduced recurrence; ~30% better survival	Bellerba et al. (2022) and Vaughan-Shaw (2020) (21, 26)
High-dose supplementation	Possibly reduces CRC mortality	RR: 0.87 for mortality (CI: 0.78–0.96)	Emmanouilidou G. et al. (2022) (20)
UVB sunlight exposure	Primary source of serum vitamin D	~90% of vitamin D synthesized through sunlight	McGregor et al. (2020) (25), Introduction
Low UV regions/dark skin	Associated with higher vitamin D deficiency, higher CRC risk	Inverse association between vitamin D and CRC	Weinstein et al. (2018) (10)
Obesity	Reduces circulating vitamin D via sequestration, worsening prognosis	Higher deficiency prevalence (PR: 33–37%)	Väyrynen et al. (2016) and Pereira-Santos (2015) (13, 14)
Magnesium Intake	Synergistic effect with vitamin D on reducing mortality	57% reduced all-cause mortality in synergy	Wesselink et al. (2020) (28)
Personalized Dosing (genetics)	May enhance response to supplementation	VDR polymorphisms affect efficacy	Pereira et al. (2024) and Lv et al. (2021) (8, 29)

## 9 Conclusion

While the protective role of vitamin D against CRC development is well-supported, its impact on CRC prognosis through supplementation remains a controversial and evolving field. Current literature suggests potential benefits, particularly in specific subgroups of patients, but highlights the need for more targeted research to address deficits in existing trials. Furthermore, while supplementation may provide marginal benefit in deficient individuals, its impact is modest compared to endogenous synthesis via UV exposure, which accounts for the majority of circulating calcitriol. The relationship between CRC, vitamin D deficiency, and obesity presents an additional layer of complexity, as changes in vitamin D metabolism secondary to obesity may affect CRC prognosis. Although conflicting data exist regarding cancer rates and mortality reduction, vitamin D supplementation has shown clinically significant improvement in survival rates, reduced recurrence risk, and enhanced quality of life in certain cases. Genetic factors also play a crucial role, with variations in genes like VDR influencing CRC risk and progression by affecting mRNA stability and gene expression regulation. Future studies should focus on optimal dosing strategies, understanding the role of genetic predispositions, and exploring the combined effects of vitamin D supplementation and weight management in improving CRC outcomes. Through these avenues, a more personalized and effective approach to CRC treatment and prevention may be achieved.
