# Correction: Vitamin D deficiency, supplementation, and colorectal cancer outcomes: interactions with obesity and risk profiles

**DOI:** 10.3389/fmed.2025.1706937

**Published:** 2025-09-29

**Authors:** Basmalah Naji, Menatalla Eltawil, Najla Nemer, Omer Abdelazim, Jayaditya D. Patil, Salim Fredericks

**Affiliations:** ^1^School of Medicine, RCSI – Medical University of Bahrain, Al Sayh, Bahrain; ^2^Department of Surgery, Yale, CT, United States; ^3^Department of Biochemistry, School of Medicine, RCSI – Medical University of Bahrain, Al Sayh, Bahrain

**Keywords:** vitamin D, colorectal cancer, VDR polymorphisms, vitamin D supplementation, obesity, cancer prognosis

Author [Basmalah Naji] was erroneously spelled as [Basmalah Ghazy].

In the published article, there was a mistake in one of the Authors' names. The Author's name for the Vitamin D Deficiency, Supplementation, & Colorectal Cancer Outcomes: Interactions with Obesity & Risk Profiles article was displayed as “Basmalah Ghazy”. The correct statement is “Basmalah Naji”.

In the published article, there was a mistake in the initials used in the Author contributions section. The initials for the Vitamin D Deficiency, Supplementation, & Colorectal Cancer Outcomes: Interactions with Obesity & Risk Profiles article was displayed as “BG”. The correct statement is “BN”.

In the published article, there was a mistake in the full citation. In the beginning of the full citation for the Vitamin D Deficiency, Supplementation, & Colorectal Cancer Outcomes: Interactions with Obesity & Risk Profiles article was displayed as “Ghazy, B.”. The correct statement is “Naji, B.”

The original version of this article has been updated.

